# Tumour Dissemination in Multiple Myeloma Disease Progression and Relapse: A Potential Therapeutic Target in High-Risk Myeloma

**DOI:** 10.3390/cancers12123643

**Published:** 2020-12-04

**Authors:** Mara N. Zeissig, Andrew C. W. Zannettino, Kate Vandyke

**Affiliations:** 1 Myeloma Research Laboratory, Faculty of Health and Medical Sciences, Adelaide Medical School, The University of Australia, Adelaide 5005, Australia; mara.zeissig@petermac.org (M.N.Z.); andrew.zannettino@adelaide.edu.au (A.C.W.Z.); 2 Precision Medicine Theme, South Australian Health and Medical Research Institute, Adelaide 5000, Australia; 3 Central Adelaide Local Health Network, Adelaide 5000, Australia; 4 Centre for Cancer Biology, University of South Australia, Adelaide 5000, Australia

**Keywords:** multiple myeloma, dissemination, metastasis

## Abstract

**Simple Summary:**

Like in solid cancers, the process of dissemination is a critical feature of disease progression in the blood cancer multiple myeloma. At diagnosis, myeloma patients have cancer that has spread throughout the bone marrow, with patients with more disseminatory myeloma having worse outcomes for their disease. In this review, we discuss the current understanding of the mechanisms that underpin the dissemination process in multiple myeloma. Furthermore, we discuss the potential for the use of therapies that target the dissemination process as a novel means of improving outcomes for multiple myeloma patients.

**Abstract:**

Multiple myeloma (MM) is a plasma cell (PC) malignancy characterised by the presence of MM PCs at multiple sites throughout the bone marrow. Increased numbers of peripheral blood MM PCs are associated with rapid disease progression, shorter time to relapse and are a feature of advanced disease. In this review, the current understanding of the process of MM PC dissemination and the extrinsic and intrinsic factors potentially driving it are addressed through analysis of patient-derived MM PCs and MM cell lines as well as mouse models of homing and dissemination. In addition, we discuss how patient cytogenetic subgroups that present with highly disseminated disease, such as t(4;14), t(14;16) and t(14;20), suggest that intrinsic properties of MM PC influence their ability to disseminate. Finally, we discuss the possibility of using therapeutic targeting of tumour dissemination to slow disease progression and prevent overt relapse.

## 1. The Role of Dissemination in the Progression of Multiple Myeloma

Multiple myeloma (MM) is an incurable haematological malignancy characterised by the uncontrolled proliferation of clonal malignant plasma cells (PCs) in the bone marrow (BM) [1]. Worldwide, MM accounts for 1% of all cancers [2], with approximately 140,000 people diagnosed with myeloma every year [3]. A characteristic feature of MM is that, at the time of diagnosis, MM PCs are present at multiple sites throughout the skeleton [1].

During initial disease development, malignant clonal PCs establish themselves in BM niches that support their growth [4]. Migration and population of these transformed clonal PCs throughout the BM leads to the development of the pre-myeloma disease stages monoclonal gammopathy of undetermined significance (MGUS) or smouldering MM and, ultimately, MM. Notably, the detection of multiple tumour lesions within the BM is a defining feature of MM, with elevated numbers of MRI-detectable lesions being an independent predictor of poor prognosis in asymptomatic [5,6,7] or symptomatic [8,9] myeloma. Furthermore, over two-thirds of newly diagnosed MM patients have detectable circulating PCs, as detected by flow cytometry [10], cytology [11] or slide-based immunofluorescence [12], with higher numbers being an independent predictor of shorter progression-free survival [13,14] and overall survival [10,11,12,13]. Notably, the association between poor outcomes and elevated clonal PCs in the peripheral blood (PB) is independent of BM tumour burden [13], suggesting an active role for circulating PCs in MM disease progression and relapse following therapy. Furthermore, the dissemination of MM PCs is a key feature of aggressive, advanced forms of MM, including in extramedullary disease and plasma cell leukaemia (PCL). PCL is characterised by the presence of very high (>20%) numbers of circulating PCs and is observed in 2–4% of newly diagnosed MM patients [15]. Extramedullary disease, characterised by the dissemination and growth of clonal PCs at soft-tissue sites, occurs in 3–5% of MM cases at diagnosis and increases to up to 20% at relapse (reviewed in Jagosky and Usmani [16]). Importantly, patients with either PCL or extramedullary disease have a poorer prognosis compared with other MM patients [15], highlighting the association between dissemination and aggressive disease.

MGUS and smouldering MM are largely asymptomatic precursor diseases characterised by the presence of clonal PCs in the BM in the absence of evidence of end organ damage that is characteristic of MM and, notably, in a lack of multiple MRI-detectable tumours [1]. Studies using next generation sequencing examining clonal evolution during disease progression from MGUS or smouldering MM to MM suggest that the genetic abnormalities leading to MM are already present at the pre-malignant stages in the majority of patients [17,18,19]. These studies suggest that the outgrowth of dominant PC clones, and their subsequent population of sites throughout the BM, is a key feature of progression to MM. In support of this, increased incidence of circulating PCs in MGUS and smouldering MM patients is associated with progression [20,21]. Taken together, these studies suggest that dissemination of clonal PCs is a key step for the progression to symptomatic disease.

In this review, we will discuss the current understanding of the mechanisms that underpin MM PC dissemination. Furthermore, we will discuss how this knowledge could be used to develop novel therapeutic strategies to delay disease progression and treat high-risk MM patients.

## 2. The Process of Dissemination in MM

Similar to the process of solid tumour metastasis, the dissemination of MM PCs is associated with a loss of their adherence to cells of the BM microenvironment that favours MM PC retention, allowing the cells to exit the niche. The tumour cells must then undergo trans-endothelial migration, mediated by chemoattractants and adhesive interactions, and intravasate (move from the BM into the bloodstream) where they are carried to a secondary site. In order to home to new BM sites, the tumour cells must arrest on the BM endothelium and extravasate (move out of the blood and into tissues), following chemotactic factors produced by BM cells. The final stage of MM PC dissemination is associated with the colonisation of new BM sites which supports tumour cell growth. The following sections will describe the molecular mechanisms that are involved in MM PC dissemination, focusing on both the intrinsic properties of the MM PCs that support their dissemination, as well as the extrinsic stimuli that drive this process.

### 2.1. Retention within the BM Stromal Niche

**Figure 1:**
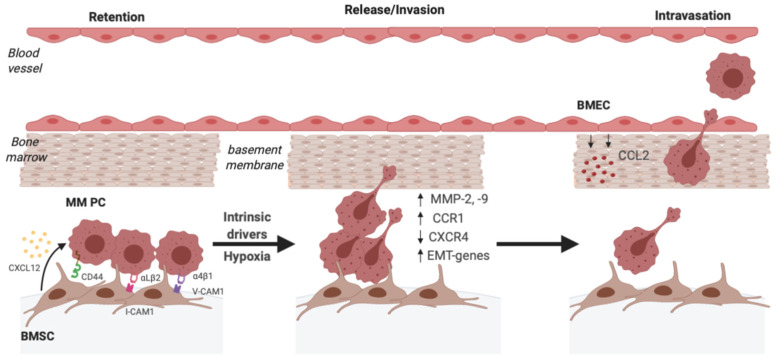
Multiple myeloma (MM) plasma cell (PC) intravasation is dependent on overcoming adhesive interactions within the bone marrow (BM) and invading the vasculature basement membrane. Release from the stromal niche is likely mediated by both external drivers such as hypoxia, and internal drivers in subclones with increased propensity to disseminate. This leads to a decrease in MM PC expression of key adhesion molecules such as α4β1, allowing the MM PCs to overcome the retention signal mediated by CXCL12 produced by bone marrow mesenchymal stromal cells (BMSCs). Hypoxic MM PCs upregulate epithelial to mesenchymal transition (EMT)-like genes, decrease their cell surface expression of CXCR4 and increase their expression of CCR1 to mediate mobilisation from the BM niche. Upregulation of matrix metalloproteins-2 and -9 then allows invasion into the vasculature basement membranes and intravasation into the peripheral circulation during dissemination to a new site.

BM mesenchymal stromal cells (BMSCs) are a critical component in the BM niche that supports the growth of MM PCs. Adhesion of MM PCs to BMSCs, and extracellular matrix (ECM) components that are secreted by these cells, is a crucial mechanism by which MM PCs are maintained within the niche (Figure 1). Binding of the integrin α4β1 (also known as very late antigen 4, VLA-4), expressed by MM PCs, to vascular cell–adhesion molecule 1 (VCAM-1) and fibronectin, expressed by BMSCs, is one of the key factors mediating the strong adhesion of MM PCs to BMSCs [22]. Notably, integrin α4β1-mediated binding to fibronectin decreases the response of MM cell lines to chemotactic factors in vitro, supporting its role in MM PC retention [23]. Additionally, the integrin αLβ2 complex (also known as lymphocyte function-associated antigen 1, LFA-1) on MM PCs mediates binding to intracellular adhesion molecule 1 (ICAM-1) on BMSCs [24]. Other adhesive factors expressed by MM PCs include CD44 variants CD44v6 and CD44v9, which mediate adhesion to BMSCs [25], and syndecan-1 (also known as CD138), which is involved in adhesion to type 1 collagen [26].

The adhesion of MM PCs to BMSCs and ECM can be enhanced by exogenous secreted factors which induce rapid cytoskeletal remodelling and conformational changes in integrins, thereby increasing adhesion [22,27,28]. The C-X-C chemokine ligand 12 (CXCL12; also known as stromal-derived factor-1 [SDF-1]), abundantly produced by BMSCs [29,30,31,32], is the ligand for the C-X-C chemokine receptor 4 (CXCR4), which is expressed universally on MM PCs from MM patients [33,34,35]. Treatment of MM PCs with CXCL12 rapidly increases MM PC adhesion to fibronectin and VCAM-1 on the surface of BMSCs, through induction of conformational changes in integrin α4β1 [22,27,28,31]. Furthermore, treatment with the CXCR4 antagonist plerixafor (also known as AMD3100) rapidly mobilises PCs into the blood [36]. Additionally, other factors may increase the adhesion of MM PCs to BMSCs by increasing expression of integrins. For example, the cytokine tumour necrosis factor alpha (TNF-α), secreted by MM PCs, acts in an autocrine fashion to increase expression of integrin α4β1 and integrin αLβ2 complexes on MM PCs, thereby increasing adhesion of MM cell lines to BMSCs in vitro [24]. In addition, the CD40 ligand (CD40L), expressed on haematopoietic cells, binds to the CD40 receptor on MM PCs and stimulates the adhesion of MM cell lines to BMSCs and fibronectin in vitro [37,38], via a mechanism which may involve upregulation of integrin expression [37]. Furthermore, B-cell activating factor (BAFF), which is expressed by BMSCs and osteoclasts, has also been shown to increase the adhesion of MM PCs to BMSCs, although the mechanism involved is unclear [39,40].

### 2.2. Release from the BM Niche

In order to be released from the BM and enter into the circulation, MM PCs must overcome the aforementioned adhesive interactions that act as a strong BM retention signal (Figure 1). While the microenvironmental stimuli that regulate the release from the BM niche are unclear, decreased expression of key factors involved in adhesion in the stromal niche may play a role. Studies analysing the expression of cell surface adhesion factors in PB PCs compared with BM PCs from MM patients have shown that circulating MM PCs express lower levels of integrin α4β1 compared with BM-resident MM PCs [41,42]. In addition, studies show that there are decreased levels of the activated form of integrin β1 in MM PCs in the PB compared with the BM in MM patients [43], suggesting that downregulation of its active form may in part facilitate release from the BM. Syndecan-1 expression has also been shown to be decreased in PB MM PCs compared with their BM counterparts [41] and MM PC syndecan-1 expression is associated with decreased dissemination in an in vivo model of MM [44]. Notably, treatment with a syndecan-1 blocking antibody rapidly induced the mobilisation of MM PCs from the BM to the PB in a syngeneic mouse model of MM [44]. Additionally, MM cell line expression of the enzyme heparanase-1, which is responsible for cleaving proteoglycans including syndecan-1 from the cell surface, significantly increased the spontaneous dissemination of MM cells in vivo, suggesting that shedding of syndecan-1 may promote dissemination [45]. Finally, PCs from PCL patients have been reported to have decreased CD40 expression compared with MGUS PCs, supporting a potential role for loss of CD40 expression in release of MM PCs from the BM niche [46].

### 2.3. Microenvironmental Control of Release from the BM Niche

Release from the BM niche may also be facilitated by signals from the micro-environment (Figure 1). The BM becomes increasingly hypoxic during MM tumour growth, with highly hypoxic regions arising within the tumour mass due to rapid tumour cell growth and abnormal blood vessel formation (reviewed by Martin et al. [47]). Moreover, the role of hypoxia in tumour progression, dissemination and angiogenesis has been demonstrated in mouse models of MM [48,49,50]. In particular, Azab and colleagues demonstrated in a mouse model of MM that MM PC hypoxia, as assessed by pimonidazole staining, strongly correlated with both BM tumour burden and numbers of circulating MM PCs [48]. Furthermore, transcriptomic analyses of paired BM and PB tumour samples from MM patients have revealed a hypoxia-related gene expression signature in MM PCs in the circulation, suggesting that hypoxia may selectively induce the mobilisation of MM PCs from the BM [51]. In addition, culturing MM cell lines in hypoxic conditions significantly reduced their adherence to BMSCs or to collagen in vitro [48,52], suggesting a mechanism whereby hypoxia may induce the release of MM PCs from the BM niche. Hypoxia has been shown to increase MM PC expression of the transcription factors Snail and Twist1 that are master regulators of the epithelial to mesenchymal transition (EMT), suggesting that, like in epithelial cancers, an EMT-like process may also be occurring in MM to allow release from the niche [48,53]. Studies by our group have demonstrated that the induction of hypoxia inducible factor HIF-2α in MM cell lines can lead to decreased response to stromal cell-derived CXCL12, which may also facilitate release from the niche. HIF-2α increases CXCL12 expression by MM cell lines [54] which, in turn, reduces CXCR4 cell-surface levels on MM cells [35], forming a feedback loop that leads to a decrease in CXCR4 signalling and a desensitisation to exogenous CXCL12 [35]. In addition, our group has demonstrated that hypoxic activation of HIF-2α leads to upregulation of the C-C chemokine receptor 1 (CCR1) in MM PCs, which may also contribute to their preferential mobilisation [35]. Notably, treatment of CCR1-positive MM cell lines with the CCR1 ligand C-C chemokine ligand 3 (CCL3; also known as macrophage inflammatory protein 1 alpha [MIP-1 α]) abrogates migration towards CXCL12 in vitro, suggesting that CCL3/CCR1 signalling can desensitise cells to exogenous CXCL12 [35]. Furthermore, either CCR1 knockout or treatment with a small molecule CCR1 inhibitor strongly inhibits the spontaneous dissemination of human MM cell lines in an intratibial model of myeloma [55]. Taken together, these studies suggest that hypoxia may mediate a disruption to the CXCR4/CXCL12 retention signal to allow MM PC release from the BM.

### 2.4. Intravasation

In order to undergo haematogenous dissemination, following release from the BM stromal niche the MM PCs need to migrate towards the vasculature, and then invade and traverse through the basement membrane and the endothelium to enter the blood stream (Figure 1). MM PCs must migrate through and invade basement membranes comprised of type IV collagen, laminin, and other ECM components. Upregulation of MM PC secretion of specific matrix metalloprotease (MMP) enzymes that degrade ECM facilitates invasion through the ECM into the vasculature (Figure 1). MMP-2 and -9, which degrade ECM components including collagen IV and laminin, have been implicated in the invasion of MM PCs [56,57].

BM endothelial cells (BMECs) are sources of secreted factors that are known chemoattractants for MM PCs, which may encourage dissemination from the primary tumour site towards the vasculature (Table 1). Conditioned media from BMECs has been shown to contain chemotactic factors for MM cell lines, including CCL2 (also known as monocyte chemotactic protein-1 [MCP-1]; ligand for CCR2), which induce chemotaxis in MM cell lines and primary MM PCs in vitro [33,58,59]. In support of this, migration of mouse 5T MM cells towards BMEC conditioned media can be blocked using an antibody against CCL2, suggesting that BMEC-derived CCL2 may promote MM PC migration towards the vasculature [58]. The production of promigratory factors by BMECs may create a promigratory stimulus that directs the migration of MM PCs from their niche towards the vasculature, allowing subsequent entry into the blood stream.

### 2.5. Extravasation and Homing to the BM

**Figure 2:**
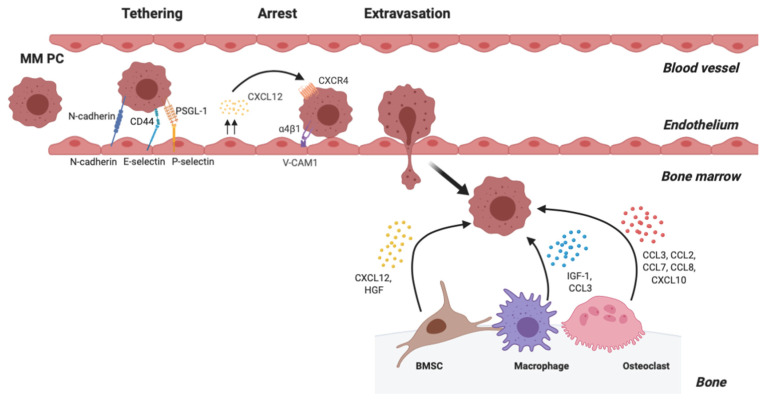
Multiple myeloma (MM) plasma cell (PC) extravasation is dependent on expression of key adhesion and chemoattractant molecules. Tethering and rolling are mediated by MM PC CD44 and P-selectin glycoprotein ligand-1 (PSGL-1) binding to E- and P-selectin, respectively, on endothelial cells), and homophilic adhesion of N-cadherin between MM PCs and endothelial cells. CXCL12, produced by the endothelium, activates MM PC-expressed integrin complex α4β1, allowing arrest through firm adhesive interactions with VCAM-1 ligand expressed on endothelial cells. Following arrest, MM PCs undergo trans-endothelial migration following chemoattractant factors expressed within the bone marrow (BM) by BM mesenchymal stromal cells (BMSCs), macrophages and osteoclasts.

Once in the blood stream, MM PCs travel in the circulation until they reach a distant site where, in response to local chemoattractant factors, they adhere to the endothelial layer and then extravasate and migrate to a new BM site (Figure 2).

MM PC adhesion to BMECs is a critical process which enables subsequent extravasation from the vasculature. In the trafficking of lymphocytes, initial reversible adhesive interactions lead to loose “tethering” of the cells to the endothelium, following which the lymphocyte rolls along the endothelium, allowing it to respond to local chemokines which provide the stimulus for extravasation and homing into the BM (Figure 2) (reviewed in Kunkel & Butcher [60]). Tethering and rolling of MM PCs is mediated by the binding of CD44 and P-selectin glycoprotein ligand-1 (PSGL-1) on MM PCs to P-selectin and hyaluronic acid, respectively, on the endothelium [61,62]. Indeed, inhibitors which prevent PSGL-1 interacting with P-selectin have been shown to result in a reduction of in vivo homing following intravenous injection of MM.1S cells [63,64]. Furthermore, treatment with an anti-CD44 antibody resulted in a decrease in 5T myeloma cell homing to the BM following intravenous injection in a mouse model [63]. Studies by our group and those of others have also implicated the homophilic adhesion molecule N-cadherin in the adhesion of MM PCs to the endothelium. shRNA knockdown of N-cadherin in human and murine MM cell lines resulted in a reduction of the adhesion of MM cell lines to BMECs under shear flow [65] without affecting transendothelial migration in vitro [65,66], suggesting a role for N-cadherin in the tethering process, but not in subsequent extravasation. In support of this, N-cadherin knockdown or an N-cadherin blocking peptide decreased the homing of MM cells to the BM following intravenous injection in mice [65,66].

Rolling along the endothelium allows the MM PCs to respond to locally produced chemokines, in particular CXCL12, which induce strong adhesive interactions through stabilising integrin-mediated adhesion to the endothelium and allowing extravasation and homing into the BM (Figure 2). Notably, studies using primary patient samples have demonstrated that CXCR4 is upregulated in MM PCs in the PB, when compared with those in the BM [28,35], potentially enabling enhanced response to local BMEC-derived CXCL12. The preferential arrest of circulating MM PCs in the BM vasculature may, at least in part, be due to the abundant expression of CXCL12 by endothelial cells in the BM (Figure 2) [29,33,67,68]. A murine MM cell line has been shown to preferentially adhere to endothelial cells from the BM, compared with those of other organs, suggesting a preference for the BM as a site of establishment [69]. Notably, Sipkins and colleagues have demonstrated that the MM cell line U266, and other B-cell lines, preferentially extravasates in the BM at specific vascular regions where endothelial expression of CXCL12 is high [68].

CXCL12 produced by BMECs has been shown to induce rapid activation of integrin α4β1 in primary MM PCs and MM cell lines, enabling arrest of the MM PCs through adhesion to BMEC VCAM-1, and subsequently enabling transendothelial migration [70]. Transendothelial migration of human myeloma cell lines in vitro [70] and in vivo [71] and primary MM PCs in vitro [70] could be completely blocked using antibodies against VCAM-1, integrin α4 or integrin β1, suggesting a role for integrin α4β1-VCAM-1 interaction in adhesion to BMECs and subsequent extravasation. In support of this, blocking of integrin α4β1 binding with an antibody delayed the in vivo homing of the human MM cell line MM.1S to the BM following intravenous injection [72]. Adhesion to the endothelium [73] and exposure to CXCL12 [74] also triggers upregulation of MM PC production of MMP9 which facilitates proteolytic degradation of the basement membrane of the endothelium [75] to further facilitate homing to the BM.

### 2.6. Establishment and Colonisation of MM PCs in a New BM Niche

Following extravasation, MM PCs further respond to locally produced chemokines and growth factors to direct their movement to their ultimate location in the BM (Figure 2). The migration of MM PCs towards specific niches in the BM may be driven by a range of chemoattractant molecules that are produced by BM cells, including BMSCs, osteoclasts and macrophages, which play an important role in the MM BM niche (Table 1). For both normal PCs and MM PCs, CXCL12 represents the predominant signal that is thought to drive homing from the PB into the BM [28,30,76,77] and subsequently also leads to MM PC retention in the BM (described in the retention within the niche section, above). CXCL12 expression is higher in the BM than in the PB [28,35], consistent with its abundant expression by BMSCs [29,30,31,32], establishing a gradient that enables homing to the BM. MM PCs have a strong migratory response towards CXCL12 [27,76], with CXCL12 inducing cytoskeletal remodelling that enables MM PC migration [28,35,70]. Blockade of CXCL12-CXCR4 binding slows homing of human MM cell lines from the PB and accumulation in the BM in vivo [28,78]. BMSCs also produce hepatocyte growth factor (HGF) [79,80,81], which further enhances the chemotactic effect of CXCL12 on MM PCs [82]. Furthermore, BMSC production of exosomes containing chemokines including CCL2, CCL3, and CXCL12 may also play a role in the migration of MM PCs towards BMSCs [83]. BMSC-derived exosomes increase the migration of the mouse myeloma cell line 5T33MM in vitro, which can be reversed with inhibitors of CXCR4 or CCR2 [83]. Insulin-like growth factor 1 (IGF-1) has also been shown to be a key promigratory (chemokinetic) and chemoattractant factor for murine [84] and human [82] MM cell lines in vitro which enhances the response to CXCL12 in vitro [82]. In the mouse 5T MM models, IGF-1 is a critical promoter of homing of MM PCs to the BM [84,85]. Notably, ablation of macrophages, an abundant source of IGF-1 in the BM, is sufficient to inhibit the homing of 5TGM1 cells to the BM [85]. Monocytes and macrophages also highly express the CCR1 ligand CCL3 [86,87], which is a promigratory factor for primary MM PCs and MM cell lines in vitro [76,87,88]. Osteoclasts are also a predominant source of a number of factors that induce MM PC migration in in vitro assays, including CCL3 [87,89], the CCR2 ligands CCL2, CCL7 (also known as monocyte chemotactic protein-3 [MCP-3]) and CCL8 (also known as monocyte chemotactic protein-2 [MCP-2]) [87,90] and the CXCR3 ligand CXCL10 (also known as interferon gamma-induced protein 10 [IP-10]) [76,91,92]. It is yet to be established whether these osteoclast-derived factors play a role in MM PC homing in an in vivo setting. Other cells of the BM microenvironment, including mesenchymal lineage cells such as adipocytes, may also play a role in the BM homing process. For example, adipocyte conditioned media induces the migration of MM cell lines in vitro [93,94,95] at least in part through expression of the chemoattractants CXCL12 and CCL2 [94].

Recent animal studies from our group [103] and those of others [104,105] have shed light on the fate of disseminating clones, and shown that the dissemination process is extremely inefficient: of the hundreds of MM cells that may reach the BM following intravenous injection, very few ultimately proliferate to form macroscopic tumours, with the remaining cells being maintained in a non-proliferative state [103,104,105]. This suggests that the BM microenvironment in which the MM PCs ultimately reside may determine whether an individual tumour cell is destined for dormancy or proliferation [104,106]. At this time, it remains unclear whether the homing of MM PCs to these niches is largely a stochastic process, or whether MM PCs are driven by specific microenvironment-derived factors to home to defined niches that ultimately determine their fate for dormancy or growth.

## 3. Intrinsic MM Characteristics that May Facilitate Dissemination

**Figure 3:**
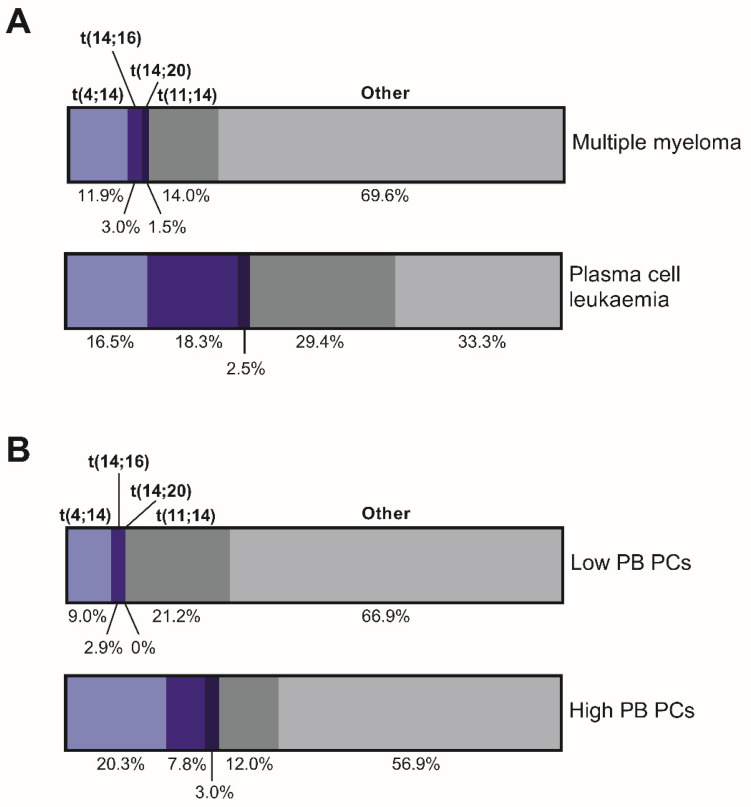
High-risk cytogenetics are associated with higher numbers of circulating multiple myeloma (MM) plasma cells (PCs) in patients. Average proportion of MM and plasma cell leukaemia (PCL) patients with common IgH translocation t(4;14), t(14;16), t(14;20) or t(11;14), or those without these translocations (other), are shown. Data is shown for patients with newly diagnosed MM or PCL (**A**) or for newly diagnosed MM patients with low or high circulating MM PC numbers (**B**). Higher incidences of high-risk cytogenetics t(4;14), t(14;16) and t(14;20) is observed in patients with PCL and in patients with high numbers of MM PCs in the peripheral blood (PB). Data is taken from [107,108,109,110,111] (**A**) and [11,14,113] (**B**).

There is growing evidence that there an association between certain chromosomal translocations that are frequently observed in MM patients, including t(14;16), t(14;20) and t(4;14), and the incidence of elevated circulating PCs or PCL [14,107,108,109,110,111,112] suggesting that these chromosomal translocations may result in an increased propensity for MM PC dissemination (Figure 3; Table 2). Additionally, t(11;14) is also highly represented in PCL [107,108,109], but is not associated with increased PB MM cell numbers in MM patients [113]; the mechanism that underlies the propensity for development of PCL in these patients remains unclear. As discussed below, there is increasing evidence for functional differences arising from t(14;16), t(14;20), and t(4;14) translocations which may underlie the propensity for dissemination observed in these genetic subtypes of MM.

### 3.1. t(14;16) and t(14;20)

The chromosomal translocations t(14;16) and t(14;20) lead to constitutive overexpression of the transcription factors MAF and MAFB, respectively. Notably, 15% of t(14;16) MM patients present with PCL, compared with 1.5% of non-t(14;16) MM patients [111]. Furthermore, the t(14;16) translocation is seen 6.5–9% of patients with elevated PB MM cells, compared with only 2–3.7% of patients with low PB MM cells [14,113], suggesting an association with increased dissemination. The relative infrequency of t(14;20) translocations has precluded statistical analysis of the association between MM PC dissemination and t(14;20); however, t(14;20) has been reported in 3% of MM patients with elevated PB PCs [14] and 2.5% of PCL patients [108], compared with 1.0–1.5% of all MM patients [14,108], suggesting a potentially increased incidence of dissemination in these patients.

Transcriptome profiling has revealed that elevation of expression of two genes, the integrin *ITGB7* and the chemokine receptor *CX3CR1*, are highly characteristic of MM patients and human MM cell lines with MAF or MAFB translocations [114,115,116,119]. Overexpression and siRNA knockdown studies have demonstrated that MAF regulates integrin β7 expression in human MM cell lines [116]. Integrin β7 can form a heterodimer with integrin αE, with the αEβ7 complex regulating adhesion to E-cadherin on the surface of BMSCs [116,120]. Blockade of E-cadherin on BMSCs [48,116] or of integrin β7 on human MM cell lines [116,120] decreases the adhesion of MM PCs to BMSCs in vitro, suggesting that this interaction may play a role in adhesion and retention in the niche. Additionally, the α4β7 integrin complex is involved in adhesion to VCAM-1 [121], suggesting a potential role for integrin β7 expression in adhesion to endothelial cells. shRNA knockdown of integrin β7 decreases the migration of human MM cell lines towards CXCL12 in vitro and, furthermore, delays BM homing following intravenous injection in vivo [120]. Elevated integrin β7 expression in MM PCs may, therefore, contribute to the increased dissemination seen in patients with aberrant MAF expression. Microarray data analyses have also shown that expression of the chemokine receptor CX3CR1 is a recurrent feature of patients with t(14;16) [114,115]. The ligand for CX3CR1, CX3CL1, can be released in a soluble form where it can act as a chemoattractant or, alternatively, can be presented in a membrane-bound form, where it facilitates cell-cell adhesion [122]. Notably, CX3CL1/CX3CR1 binding has been implicated in playing a key role in the transendothelial migration of lymphocytes by increasing endothelial cell adhesion and migration towards chemoattractants [123]. While the functional role for CX3CR1 in MM is unclear, the human MM cell line RPMI-8226 has been shown to bind to CX3CL1 in vitro under shear flow, suggesting that it may play a role in adhesion to endothelial cells in MM [124]. Gene expression profiling studies have also identified elevated expression of *IGF1R* and *CCR1*, the receptors for the MM PC chemoattractants IGF-1 and CCL3, respectively, are also elevated in t(14;16) human MM cell lines and primary MM PCs [99,116]. The expression of these key promigratory receptors may also, therefore, play a role in the MM PC dissemination in t(14;16) patients.

### 3.2. t(4;14)

The chromosomal translocation t(4;14), which leads to constitutive overexpression of the histone methyltransferase NSD2 (also known as WHSC1 and MMSET), is also associated with high risk disease and increased dissemination in MM patients. The incidence of patients with t(4;14) is 2- to 4-fold higher in patients with elevated circulating PCs, compared with those with low circulating PCs [11,14,113,125]. Additionally, there is some evidence for an increase in the incidence of t(4;14) in PCL patients [109] and t(4;14) MM is also associated with an increased incidence of extramedullary PC tumours [126].

Notably, the t(4;14) translocation is associated with an EMT-like gene expression signature in MM PCs, characterised by upregulation of mesenchymal genes, including those for N-cadherin and vimentin, and the transcription factor Twist-1 [53,66,117,118]. N-cadherin knockdown studies in MM cell lines have shown that N-cadherin is important in the homing of MM PCs from the vasculature to the BM [65,66]. In addition, studies from our group have shown that overexpression of Twist-1 increases MM PCs dissemination in an intratibial model of MM in vivo [53]. Like t(14;16) patients, tumour cells from t(4;14) patients and MM cell lines have been shown to have increased expression of *IGF1R* and *CCR1* receptors [99,100,116] which may also contribute to the increased propensity for dissemination of t(4;14) MM PC.

### 3.3. Subclonal Heterogeneity and Dissemination

There is evidence to suggest that, in addition to these chromosomal translocations, other secondary chromosomal abnormalities and mutations may increase the ability of individual subclones to disseminate [41,127,128,129]. Using FISH or whole exome sequencing on paired BM and PB PC samples from MM patients, several studies have found that in some instances subclones present in the BM were not present in the PB, and vice versa [41,128,129]. However, it remains to be seen whether the subclonal differences observed between the BM and PB in MM patients are due to selective microenvironmental pressures, such as hypoxia or other as yet uncharacterised stimuli, that drive the efflux of a subclone in a particular microenvironment, or if certain secondary copy number changes or SNVs may increase the propensity for dissemination in MM, leading to an increased likelihood for dissemination of particular subclones. Furthermore, it is also possible that the observed subclonal differences may be due to the inability of single-site BM biopsy to capture the spatial heterogeneity seen in this disease [130].

There is some evidence that points to mechanisms whereby certain subclones could have an increased propensity for dissemination. Deletion of chromosome 17p13 [del(17p)] occurs in approximately 10% of newly diagnosed MM patients [109,110,113,131]. Missense mutations in *TP53*, on 17p, are seen in approximately 19% of patients with del(17p), leading to a complete inactivation of functional p53 [132]. There is some evidence for an increased incidence of del(17p) in PCL patients [109], although this has not been reported in MM patients with elevated PB PC [11,113]. In a study utilising whole exome sequencing of paired BM and PB tumour samples from 4 patients, Manier et al. identified one patient with 17p loss across the BM and PB sample with a *TP53* missense mutation which was only detectable in the PB sample, suggesting a potential association between loss of p53 function and increased the propensity for dissemination [129]. In support of this, siRNA-mediated knockdown of p53 in NCI-H929 cells has been shown to increase invasion through Matrigel and decreased adhesion to BMSCs in vitro [133]. Although more studies are needed, these data suggest a potential role for loss of p53 in the dissemination of MM PC.

## 4. Future Perspectives

One of the defining features of symptomatic MM is the presence of MM tumours at multiple sites in the BM [1]. In over two-thirds of newly diagnosed MM patients, circulating MM PCs are detectable in the PB [10,11,12], with higher numbers being associated with poorer overall survival [10,11,12,13]. Furthermore, highly disseminated disease, as characterised by elevated circulating MM PCs and extramedullary tumours, is a feature of advanced disease and is associated with poorer overall survival [14,111,134,135]. There is also evidence to suggest that dissemination of therapy-resistant clones leads to shorter time to relapse, with higher numbers of circulating tumour cells at baseline being indicative of shorter progression-free survival, regardless of the therapy used [13,14,136,137].

These studies suggest that therapies which target key processes involved in MM PC dissemination may be useful to delay disease progression or as a maintenance therapy to extend progression-free survival following frontline therapy. To this end, studies suggest that therapeutic targeting adhesion molecules or chemokine receptors may be able to inhibit the ability of MM cells to egress from or to home to the BM. For example, selective blocking of adhesion molecules involved in the adhesion to endothelium, such as integrins and N-cadherin, have been shown to inhibit homing and thereby decrease subsequent tumour burden following intravenous injection of tumour cells in mouse models of MM [63,65]. Additionally, blockade of CXCL12/CXCR4 with plerixafor or a CXCL12-targeting L-RNA aptamer has been shown to decrease BM tumour establishment following intravenous injection in mouse models of MM [28,78]. However, paradoxically, CXCL12/CXCR4 blockade also mobilises MM PC from the BM niche in tumour bearing mice [36,78] and in patients [138], which could inadvertently increase tumour dissemination. Recent studies from our group have shown that treatment with a small molecule inhibitor of the chemokine receptor CCR1, CCX9588, can prevent the dissemination of MM PCs from the BM in intratibial MM models in vivo [55]. As far as we are aware, this is the first preclinical demonstration of a therapeutic intervention that can inhibit spontaneous dissemination of MM cells in vivo. However, it remains to be seen whether therapeutic targeting of dissemination will translate to a beneficial impact on subsequent disease progression and, to date, no anti-dissemination treatment strategies have reached clinical trial for MM.

An alternative approach could be to therapeutically target hypoxic MM cells before they disseminate. Evofosfamide (TH-302), a DNA alkylator designed to be selectively activated under hypoxic conditions, has been shown to cause apoptosis of MM cell lines when cultured under hypoxia in vitro and to inhibit MM tumour development in vivo [50,139]. In a Phase I/II trial in relapsed/refractory myeloma patients, treatment with evofosfamide in combination with dexamethasone, with or without bortezomib, was associated with a modest response rate, with partial or better response observed in 12% of these heavily pre-treated patients [140]. Taken together, these studies suggest that targeting of hypoxic MM PCs could be investigated as a therapeutic strategy to prevent MM PC dissemination.

## 5. Conclusions

In conclusion, this review has highlighted the current understanding of the processes involved in MM dissemination. Some key outstanding questions include: Are there environmental factors other than hypoxia that drive dissemination/mobilisation? Is underlying subclonal heterogeneity leading to mobilisation of specific individual subclones during dissemination? What are the features of the BM microenvironment that support the homing and subsequent growth of MM PCs at specific niches? A greater understanding the process of MM PC dissemination may allow the identification of novel therapeutic targets that could improve outcomes for MM patients, especially for the prevention of disease progression and relapse in high-risk patients.

## Figures and Tables

**Table 1:** Receptors and secreted factors involved in the dissemination process in multiple myeloma.

Receptor on MM PCs		Pro-Disseminatory Factor	Predominant Source in The Myeloma BM		Suggested Role in Dissemination	
CXCR4	[33,34,35]	CXCL12	Hypoxic MM PCs	[35,54]	Mobilisation of MM PCs from the BM	[35,96]
			Endothelial cells	[29,33,67,68]	Arrest of circulating MM PCs in the BM vasculature	[33,67,68,69]
			BMSCs	[29,30,31,32]	Migration and homing to BM niches	[27,28,76,77]
CCR1	[34,35,55]	CCL3	MM PCs	[35,89,97,98]	Mobilisation of MM PCs from the BM	[35,55]
			Osteoclasts, macrophages	[86,87,89]	Migration and homing to BM niches	[87,88,89]
CCR2	[33,34,87]	CCL2	Endothelial cells	[33,58]	Migration towards BMECs	[33,58,59]
			BMSCs, osteoclasts, adipocytes	[32,81,87,90,94]	Migration and homing to BM niches	[33,58]
		CCL7, CCL8	Osteoclasts	[87]	Migration and homing to BM niches	[90]
IGF-1R	[99,100]	IGF-1	Macrophages	[85,87,101]	Migration and homing to BM niches	[84,85]
Met	[100,102]	HGF	BMSCs	[79,80,81]	Synergises with CXCL12 to increase MM PC migration	[82]

**Table 2:** Chromosomal translocations associated with increased dissemination in multiple myeloma (MM) and plasma cell leukaemia (PCL).

Chromosomal Translocation	Driver Gene	Association with Increased Dissemination ^1^	Putative Pro-Disseminatory Factors	
t(14;16) and t(14;20)	*MAF*/*MAFB*	Increased incidence in PCL and high-PB PC MM	Integrin β7	[102,103,104,105]
			CX3CR1	[114,115]
			IGF-1R	[99,116]
			CCR1	[99,116]
t(4;14)	*NSD2*	Increased incidence in PCL and high-PB PC MM	EMT-like gene signature (including *CDH2*, *VIM* and *TWIST1*)	[53,66,117,118]
			IGF-1R	[99,100,116]
			CCR1	[99,100,116]
t(11;14)	*CCND1*	Increased incidence in PCL	Unclear	-

^1^ See Figure 3.
